# A comprehensive approach for correcting voxel‐wise b‐value errors in diffusion MRI

**DOI:** 10.1002/mrm.28078

**Published:** 2019-12-16

**Authors:** Yoojin Lee, Adam O. Kettinger, Bertram Jakob Wilm, Ralf Deichmann, Nikolaus Weiskopf, Christian Lambert, Klaas Paul Pruessmann, Zoltan Nagy

**Affiliations:** ^1^ Laboratory for Social and Neural Systems Research (SNS Lab) University of Zurich Zurich Switzerland; ^2^ Institute for Biomedical Engineering ETH Zurich and University of Zurich Zurich Switzerland; ^3^ Brain Imaging Centre Research Centre for Natural Sciences Budapest Hungary; ^4^ Department of Nuclear Techniques Budapest University of Technology and Economics Budapest Hungary; ^5^ Brain Imaging Centre Goethe University Frankfurt Germany; ^6^ Wellcome Centre for Human Neuroimaging UCL Queen Square Institute of Neurology London United Kingdom; ^7^ Department of Neurophysics Max Planck Institute for Human Cognitive and Brain Sciences Leipzig Germany

**Keywords:** b‐value, diffusion gradient, diffusion MRI, diffusion tensor, gradient nonlinearity

## Abstract

**Purpose:**

In diffusion MRI, the actual b‐value played out on the scanner may deviate from the nominal value due to magnetic field imperfections. A simple image‐based correction method for this problem is presented.

**Methods:**

The apparent diffusion constant (ADC) of a water phantom was measured voxel‐wise along 64 diffusion directions at b = 1000 s/mm^2^. The true diffusion constant of water was estimated, considering the phantom temperature. A voxel‐wise correction factor, providing an effective b‐value including any magnetic field deviations, was determined for each diffusion direction by relating the measured ADC to the true diffusion constant. To test the method, the measured b‐value map was used to calculate the corrected voxel‐wise ADC for additionally acquired diffusion data sets on the same water phantom and data sets acquired on a small water phantom at three different positions. Diffusion tensor was estimated by applying the measured b‐value map to phantom and in vivo data sets.

**Results:**

The b‐value‐corrected ADC maps of the phantom showed the expected spatial uniformity as well as a marked improvement in consistency across diffusion directions. The b‐value correction for the brain data resulted in a 5.8% and 5.5% decrease in mean diffusivity and angular differences of the primary diffusion direction of 2.71° and 0.73° inside gray and white matter, respectively.

**Conclusion:**

The actual b‐value deviates significantly from its nominal setting, leading to a spatially variable error in the common diffusion outcome measures. The suggested method measures and corrects these artifacts.

## 1 INTRODUCTION

Diffusion MRI provides important information related to the microstructure of brain tissue and has been shown to be useful in both clinical diagnostics^1^, ^2^ and basic scientific research.^3^ In the early 1990s, diffusion tensor imaging (DTI)^4^ has emerged. DTI uses at least 6 diffusion‐weighted images with different diffusion gradient directions to derive quantitative measures of the apparent diffusion coefficient (ADC), the fractional anisotropy (FA), and structural orientations of the underlying white matter (WM) tissue.^5^ Further advances in data acquisition and processing enabled a non‐invasive delineation of the WM fiber pathways in the brain.^6^, ^7^, ^8^ Due to its sensitivity to the cellular architecture of tissue, diffusion MRI is a vital tool for in vivo microstructural imaging, relating microscopic tissue properties (e.g., neurite density, fiber orientation distribution, axon diameter) to the voxel‐wise MR signals.^9^

These techniques apply strong diffusion‐sensitizing gradient pulses along several directions, assuming a b‐value that is constant across space and all diffusion gradient directions to estimate the diffusion tensor parameters and/or fiber tracts. However, actual b‐values may deviate from the nominal b‐value in a spatially variable manner, due to gradient nonlinearities, imaging gradient interactions, concomitant fields, eddy currents affecting the diffusion‐encoding gradients, and gradient miscalibration. Given the quadratic dependence of the b‐value on the gradient strength, even relatively small gradient deviations may lead to substantial errors in b‐value, yielding erroneous values of voxel‐wise diffusion measures such as ADC and FA and may cause problems in determining the fiber orientations.^10^

Several methods have been proposed to correct for the erroneous b‐values originating from one or a combination of the aforementioned sources. A simple and straight‐forward approach is to account for contributions of the imaging gradients in the b‐value calculation. However, this approach needs a full description of the applied pulse sequence for each diffusion gradient direction, followed by an analytical or numerical calculation of b‐value for the entire sequence.^11^, ^12^ For the compensation of diffusion gradient nonlinearity effects, a correction scheme has been proposed, relying on full spatial mapping of the magnetic fields generated by each gradient coil with spherical harmonic basis functions.^13^, ^14^ The impact of failing to make corrections for the spatial nonuniformity of the b‐value caused by gradient nonlinearity effects in diffusion imaging has been demonstrated recently^15^ with Human Connectome Project data.^16^ In addition, prospective^17^ and retrospective^18^ correction approaches have been suggested to mitigate the effect of concomitant fields associated with diffusion‐sensitizing gradients that are positioned non‐symmetrically around an RF refocusing pulse. Finally, a gradient miscalibration issue has been identified and corrected by measuring the miscalibration factor for the 6 principal gradient directions (±x, ±y, and ±z) and then rescaling the gradient amplitudes.^19^ Rather than addressing each possible source of deviations separately, it has been suggested to measure deviations of the applied diffusion gradients together, regardless of their causes, using a water phantom and to correct for these deviations in a retrospective manner using a linearized model.^20^

In this work, a comprehensive, model‐free, image‐based method is proposed for determining voxel‐wise spatially dependent b‐value deviations. Although this method does not deal with the related issue of deviations in diffusion direction, it is demonstrated for a single‐shell diffusion data set (b = 1000 s/mm^2^), the results of which can be subsequently applied to phantom and in vivo data sets, yielding improved results of the DTI data analysis.

## 2 METHODS

### 2.1 Theory

The ADC map for a given diffusion gradient direction is calculated voxel‐wise using a reference image (S_0_) and a diffusion‐weighted image (S):

(1) $$\text{ADC}\;=\;\text{ln}(S_{0}/S)/b$$

where b is the b‐value used in the experiment.^21^ Using the nominal b‐value (b^nom^) despite the fact that the actual measured S_0_ ($S_{0}^{\text{meas}}$) and S (S^meas^) are affected by multiple factors (e.g., gradient nonlinearities, imaging gradients, concomitant fields, eddy currents, gradient miscalibrations) yields an erroneous ADC map (ADC^err^):

(2) $${\text{ADC}}^{\text{err}}\;=\;\text{ln}\left(S_{0}^{\text{meas}}/S^{\text{meas}}\right)/b^{\text{nom}}.$$

If an effective b‐value map (b^eff^) is defined as the set of voxel‐wise b‐values that consider all effects yielding discrepancies between the measured and the expected signal, the true ADC value can still be obtained as follows:

(3) $${\text{ADC}}^{\text{true}}\;=\;\text{ln}\left(S_{0}^{\text{meas}}/S^{\text{meas}}\right)/b^{\text{eff}}.$$

Rearranging Equations 2 and 3 yields

(4) $${c\;=\;b}^{\text{eff}}/b^{\text{nom}}{\;=\;\text{ADC}}^{\text{err}}/{\text{ADC}}^{\text{true}}.$$

Provided the true ADC (ADC^true^) is known, the correction factor (*c*) can be calculated with Equation 4 and subsequently used for b‐value correction.

### 2.2 Data acquisition

All scans were performed on a 3T Philips Achieva scanner (Philips Healthcare, Best, The Netherlands), equipped with an 8‐channel head coil and an RF body transmit coil. The DTI sequence used was based on the Stejskal‐Tanner diffusion‐encoding scheme.^21^ Four separate experiments were performed. In each experiment, the center of the imaging volume coincided with the isocenter of the magnet.

#### 2.2.1 Effective b‐value map (Experiment_1)

For the estimation of a voxel‐wise effective b‐value map (b^eff^), a large water phantom with an inner diameter of 19 cm was placed at the center of the head coil and scanned 11 times repeatedly with a high angular resolution diffusion imaging (HARDI) sequence.^22^ The first 10 data sets were used to obtain the b^eff^ map. The 11th data set was analyzed using the b^eff^ map calculated from the 10 repetitions for validation of the proposed method. The HARDI data set consisted of 2 b_0_ images (b = 0 s/mm^2^) and 64 diffusion‐weighted images (b = 1000 s/mm^2^), with the diffusion directions distributed evenly over a hemisphere. Imaging parameters for the HARDI sequence were as follows: voxel size = 1.7 mm isotropic, volume TR = 30 seconds, TE = 90 ms, sensitivity encoding (SENSE) acceleration factor = 2, field‐of‐view (FOV) = 205 × 205 × 156 mm^3^, number of slices = 92, no slice gap, slice acquisition order = interleaved, and scan time = 33.5 minutes. A long TR was chosen deliberately to avoid possible variations in the performance of the gradient or static shim system that may lead to Larmor frequency shifts. Before each HARDI scan, gradient‐echo images at 5 different TEs (TE1/ΔTE = 4.6/1.15 ms) were acquired to estimate a B_o_ field map.

#### 2.2.2 Stability of the effective b‐value map (Experiment_2)

To test the stability of the b^eff^ map over time, one additional HARDI data set was acquired 6 months later but with the same imaging sequence described in Experiment_1.

#### 2.2.3 Noise distribution of diffusion‐weighted signal (Experiment_3)

To ascertain whether the b^eff^ map measured with a water phantom was not biased due to Rician noise, diffusion‐weighted images were repeatedly acquired 217 times for one of the 64 diffusion directions with the same imaging protocol as in Experiment_1. The voxel‐wise signal‐to‐noise ratio (SNR) map was then evaluated by dividing the mean by the standard deviation across 217 diffusion‐weighted images.

#### 2.2.4 Validation on phantom (Experiment_4)

In addition, a smaller water phantom with an inner diameter of 10 cm was scanned with a DTI sequence that included 6 of the 64 diffusion directions used in Experiment_1. The small phantom was scanned at 3 different positions inside the head coil, located at a distance of approximately 5 cm from the center of the head coil along left, anterior, and superior directions. All other acquisition parameters were the same as for the HARDI sequence described previously, except for number of slices = 72, volume TR = 11 seconds, and scan time = 1.3 minutes. As in Experiment_1 a B_o_ field map was also acquired before each DTI scan.

#### 2.2.5 Effective b‐value map at several nominal b‐values (Experiment_5)

To investigate the influence of the nominal b‐value on the b^eff^ map, additional DTI data sets were collected on the large water phantom placed at the center of the head coil with b = 500 s/mm^2^ and 1000 s/mm^2^. The acquisition parameters were identical to Experiment_1, except for number of repetitions = 5 and TE = 79 ms for the b‐value of 500 s/mm^2^. The same 6 diffusion directions as in Experiment_4 were used. Twice as many repetitions for the measurement with b = 1000 s/mm^2^ were performed in order to balance the reduction in SNR. The acquisition was performed interleaved (i.e., one acquisition block consisted of one b = 500 s/mm^2^ scan and two b = 1000 s/mm^2^ scans) and this acquisition block was repeated 5 times. A B_o_ field map was acquired for each acquisition block. Additionally, for one of the diffusion directions, the b^eff^ maps were acquired with nominal b‐values between b = 300 s/mm^2^ and 1000 s/mm^2^ and a step size of 100 s/mm^2^.

#### 2.2.6 In vivo validation (Experiment_6)

One healthy male volunteer was scanned in accordance with local ethics guidelines after giving signed written informed consent. A B_o_ map and a HARDI data set with 64 diffusion directions were collected for the DTI analysis. The same HARDI sequence as for the evaluation of the b^eff^ map (Experiment_1) was used with identical parameters except for volume TR = 11 seconds, number of slices = 72, and scan time = 12.3 minutes.

#### 2.2.7 Concurrent magnetic field monitoring

For all acquisitions in the previously described 6 experiments, magnetic field variations were monitored during the readout period of the sequence using a magnetic field camera setup (Skope Magnetic Resonance Technologies, Zurich, Switzerland) to minimize image artifacts (e.g., global and local image distortions and echo‐planar‐imaging (EPI) ghosts) from undesirable field perturbations such as eddy currents affecting the readout gradients, concomitant fields, and gradient delays. The 16 transmit/receive ^19^F NMR probes^23^ distributed around the head were integrated into the head coil with the help of a 3D printed insert.^24^, ^25^ The dynamic field evolution up to the third spatial order of the spherical harmonics was obtained for the diffusion acquisitions to address the long‐term higher‐order eddy currents that arise from the diffusion‐sensitizing gradients.^26^ The information on the field dynamics together with the static B_o_ field map were exploited in the off‐line reconstruction to jointly correct for the static and dynamic field perturbation effects.^27^, ^28^ In the off‐line reconstruction, no filtering was applied in k‐space or image space to minimize the loss of resolution.

#### 2.2.8 Temperature monitoring

For estimation of the true ADC of the water phantom, which is required in Equation 4, the temperature was measured every 3 seconds during the diffusion experiment with a fiber optic temperature sensor (Opsens Solutions, Quebec, Canada) placed on the surface of the water phantoms. The ADC was then calculated according to published data^29^ using the mean temperature over the course of the diffusion scan. To ascertain that the temperature of water can be reliably estimated from a measurement on the surface of the phantom, we placed a water bottle inside the scanner and alternately and repeatedly measured the temperature of both the water and the surface of the water bottle during 5 minutes  HARDI scans.

### 2.3 B‐value correction factor

The b‐value correction map (i.e., *c* in Equation 4) was computed from the first 10 repetitions of Experiment_1, using custom‐made functions written in MATLAB (The MathWorks, Natick, MA): First, an erroneous ADC map (ADC^err^) was calculated using Equation 2 for each diffusion direction and repetition. Here, the averaged b_0_ image was used. Then, a b‐value correction map was obtained by dividing the ADC^err^ maps by the true diffusion coefficient (ADC^true^) estimated separately for each repetition from the measured temperature.^29^ The 10 b‐value correction maps were subsequently averaged to improve SNR, followed by masking, to exclude voxels without relevant signal (i.e., outside the phantom where the resulting maps are meaningless). Assuming a smoothly varying b‐value correction map, the masked mean b‐value correction map was smoothed with a 3D Gaussian kernel with a 3.4‐mm^3^ standard deviation to further reduce the noise level. To avoid an error near the edges of the phantom introduced by including the voxels outside the phantom, both the masked mean b‐value correction map and the mask itself were smoothed. When the resultant smoothed map was then divided by the smoothed mask, the effect of voxels containing zeros outside the mask were compensated.^30^ With the b‐value correction map calculated from the first 10 repetitions of Experiment_1, the b‐value errors were corrected in the 11th HARDI data set from Experiment_1 as well as the HARDI data set from Experimen_2, the 3 DTI data sets from Experiment_4, and the in vivo data set from Experiment_6.

To numerically simulate the effects of the gradient nonlinearities only, the b‐value map was calculated based on the spatial maps of the magnetic fields generated by x‐, y‐, and z‐gradient coils according to Bammer et al.^13^ In brief, the magnetic field distribution for each gradient coil was obtained using an 11th‐order spherical harmonic expansion with all even terms set to zero. Subsequently, the gradient fields for each axis were determined by calculating the spatial derivative with respect to the corresponding axis (e.g., the spatial derivative of the magnetic field map generated by the x‐gradient coil with respect to the x‐axis). Based on this information, the gradient strengths were obtained for the 64 diffusion directions used in the HARDI sequence. The square of the gradient strength divided by the nominal gradient strength served as the b‐value correction map induced by the gradient nonlinearities.

Two separate b‐value correction maps were calculated from the b = 500 s/mm^2^ and 1000 s/mm^2^ data sets in Experiment_5 analogously to that in Experiment_1. The influence of the imaging gradients on the b‐value map was also evaluated for each of the 6 diffusion directions used in Experiment_5. To achieve this, the sequence diagrams for all 6 diffusion directions were exported from the scanner, and the spatially uniform b‐value was calculated based on the following equation^31^:

(5) $$b\;=\;\int_{0}^{\text{TE}}k\left(\tau \right)\cdot k\left(\tau \right)d\tau$$

with

$$k\left(t\right)=\gamma \int_{0}^{t}G\left(\tau \right)d\tau$$

where **G**(t) = [G_x_(t) G_y_(t) G_z_(t)]^T^ is the vector consisting of gradient waveforms for the x‐, y‐, and z‐axis. The difference between the b‐value calculated from Equation 5 and the nominal b‐value represents the bias caused by the imaging gradients. The b‐value map without the effects of the imaging gradients was obtained by subtracting this bias from the measured effective b‐value map (b^eff^ in Equation 4).

### 2.4 Diffusion tensor analysis with the voxel‐wise b‐value

Using *dtifit* from FSL version 5.0.11 (http://fsl.fmrib.ox.ac.uk/fsl), the diffusion tensor was estimated on the HARDI data set of the large water phantom from Experiment_1 by applying the ordinary linear least‐squares approach^4^ for each voxel within the phantom. *Dtifit* was performed twice, using the two different b‐value approaches (i.e., once with a nominal b‐value for every voxel and once with the voxel‐wise effective b‐value obtained from the data collected on the large water phantom). To exploit the unique b‐value for each voxel in the phantom, a customized preprocessing pipeline was designed in MATLAB that served as a wrapper for the FSL libraries. A temporary diffusion image (dimension 1 × 1 × 1 × 65) was created for each voxel in the phantom. *dtifit* was performed on this one‐voxel image with the corresponding effective b‐value. The outputs from FSL for each voxel were then read back into the full volumes.

The same diffusion tensor analysis described previously was performed on the in vivo HARDI data set from Experiment_6. To extract the brain area, skull stripping was performed using SPM12 (http://www.fil.ion.ucl.ac.uk/spm). For this purpose, the b_0_ image was segmented into gray matter (GM), WM, and cerebrospinal fluid tissue classes. The resulting tissue maps were summed and binarized with a threshold of 0.1 to generate the brain mask. Subsequently, the diffusion tensor was estimated voxel‐wise within this brain mask.

## 3 RESULTS

**Figure 1:**
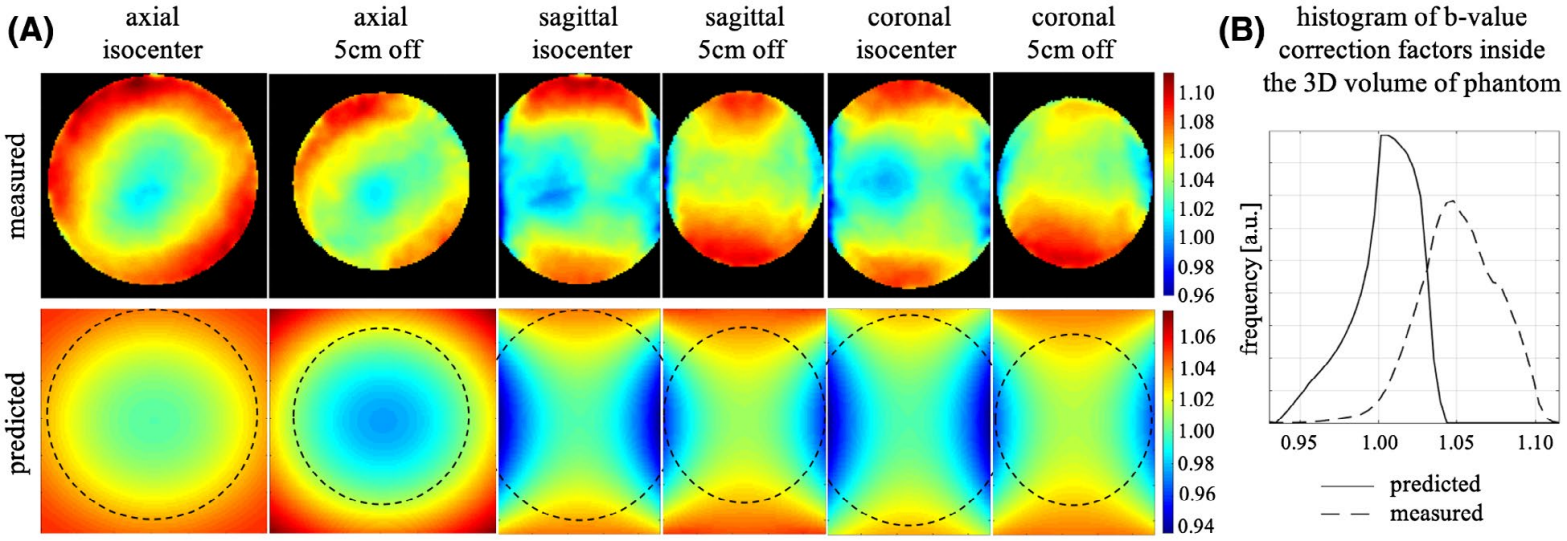
A, Axial (first and second columns), sagittal (third and fourth columns), and coronal (fifth and sixth columns) sections of the measured b‐value correction map (top row) and the predicted b‐value correction map obtained from the knowledge of the magnetic field produced by the gradient coils (bottom row), both at the isocenter (first, third, and fifth columns) and 5 cm away from the isocenter along the axis perpendicular to the respective image plane (second, fourth, and sixth columns) for 1 of the 64 diffusion directions [0.60, −0.79, −0.13], where x, y, and z correspond to left–right, anterior–posterior, and head–foot directions, respectively. The dotted circles in the bottom row represent the position of the water phantom in the measured b‐value correction map. Note the different intensity scales for the two b‐value correction maps. B, Histograms of the measured (dashed line) and predicted (solid line) b‐value correction factors across the 3D volume of the water phantom

Figure 1A shows both the measured (top) b‐value map from Experiment_1 and the predicted (bottom) b‐value correction map based on a spherical harmonic expansion.^13^ Overall, the predictions provided a pattern similar to the measured b‐value correction maps, albeit the latter captured more spatial detail and showed larger deviations from the nominal b‐value (please see also the histograms in Figure 1B and note the different color bars for the two maps). This discrepancy is most probably due to the fact that the predictions consider only one source of deviation (i.e., diffusion gradient nonlinearities), ignoring all other effects (e.g., imaging gradients, concomitant fields).

The mean temperatures of water and the surface of the water bottle were 24.88° and 24.74°, respectively, yielding a negligible 0.36% difference in diffusion coefficient.

Only 0.02% of voxels had an SNR < 2 (i.e. Rician noise distribution^32^) within the large water phantom in Experiment_3. Furthermore, a Kolmogorov‐Smirnoff test was performed on the 217 diffusion‐weighted image intensities at each voxel (α = 0.01) to ascertain that the noise distribution was Gaussian.^33^ Again, only 0.02% of the voxels exhibited non‐Gaussian distribution.

**Figure 2:**
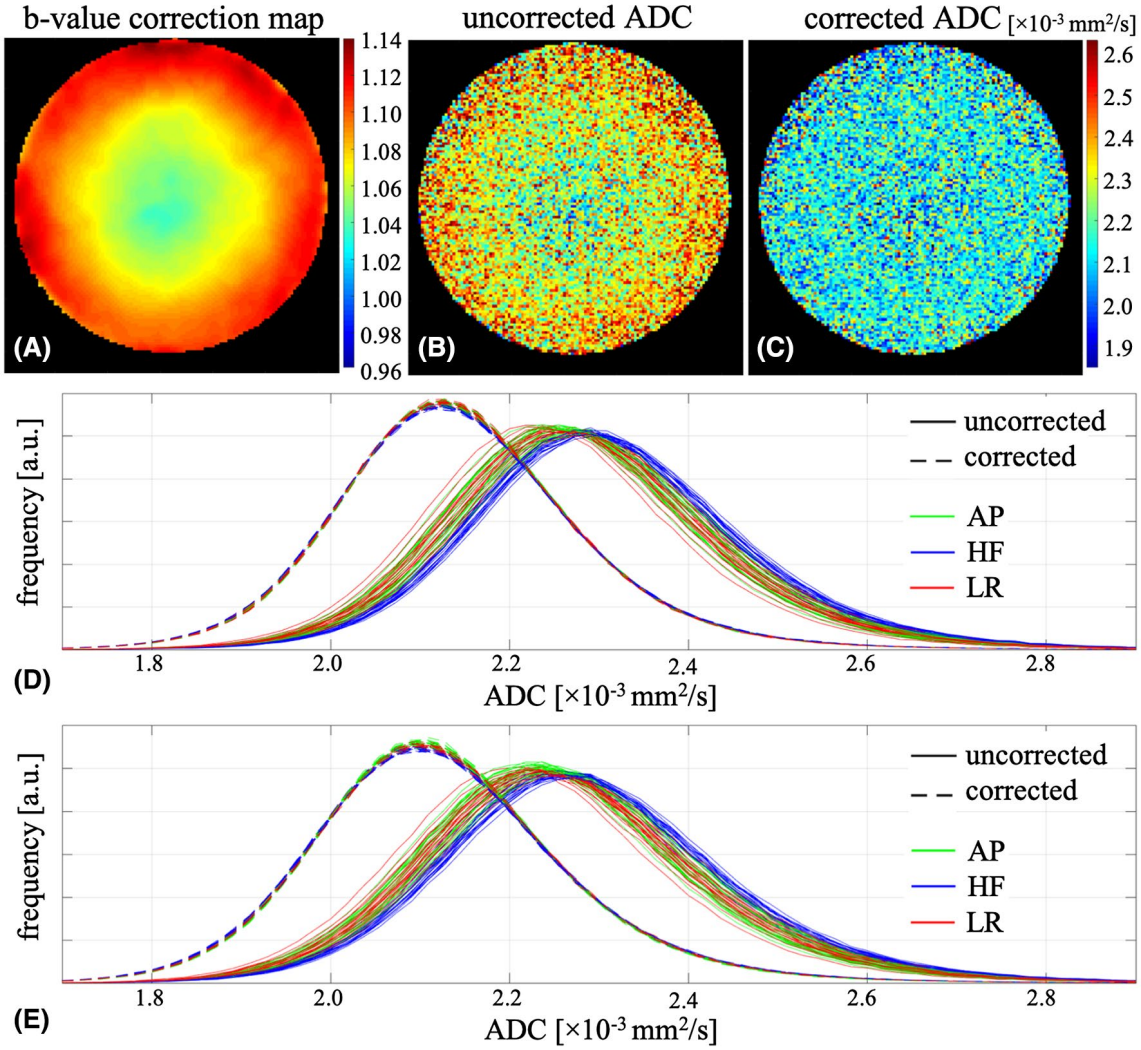
A, Central axial slice of the measured b‐value correction map for the diffusion direction of [−0.32, 0.13, −0.94]. B,C, The ADC map of the same slice and diffusion direction as in (A), without (B) and with (C) voxel‐wise b‐value correction. D,E, Histograms of the ADC values inside the water phantom for all 64 diffusion directions with (dashed lines) and without (solid lines) voxel‐wise b‐value correction for the HARDI data sets from Experiment_1 (D) and Experiment_2 (E). Note that the HARDI data set from Experiment_2 was acquired about 6 months after the measurement of the b‐value correction map. The diffusion directions that have the largest component along anterior–posterior (AP), head–foot (HF), and left‐right (LR) directions are shown as green, blue and red, respectively

Using the measured b‐value correction map from the first 10 repetitions in Experiment_1 (Figure 2A) to calculate an ADC map from the 11th HARDI data set made a marked difference. Compared to using the nominal b‐value (Figure 2B), the corrected ADC map (Figure 2C) had the expected smaller and more spatially uniform diffusion constant. Moreover, histograms of ADC values for voxels covering the whole phantom showed improved correspondence across the 64 diffusion directions after the correction (dashed lines in Figure 2D) compared with the histograms of uncorrected ADC values (solid lines in Figure 2D). The voxel‐wise b‐value correction worked similarly well for the additional HARDI data set from Experiment_2 acquired about 6 months apart from the b‐value correction map (Figure 2E).

**Figure 3:**
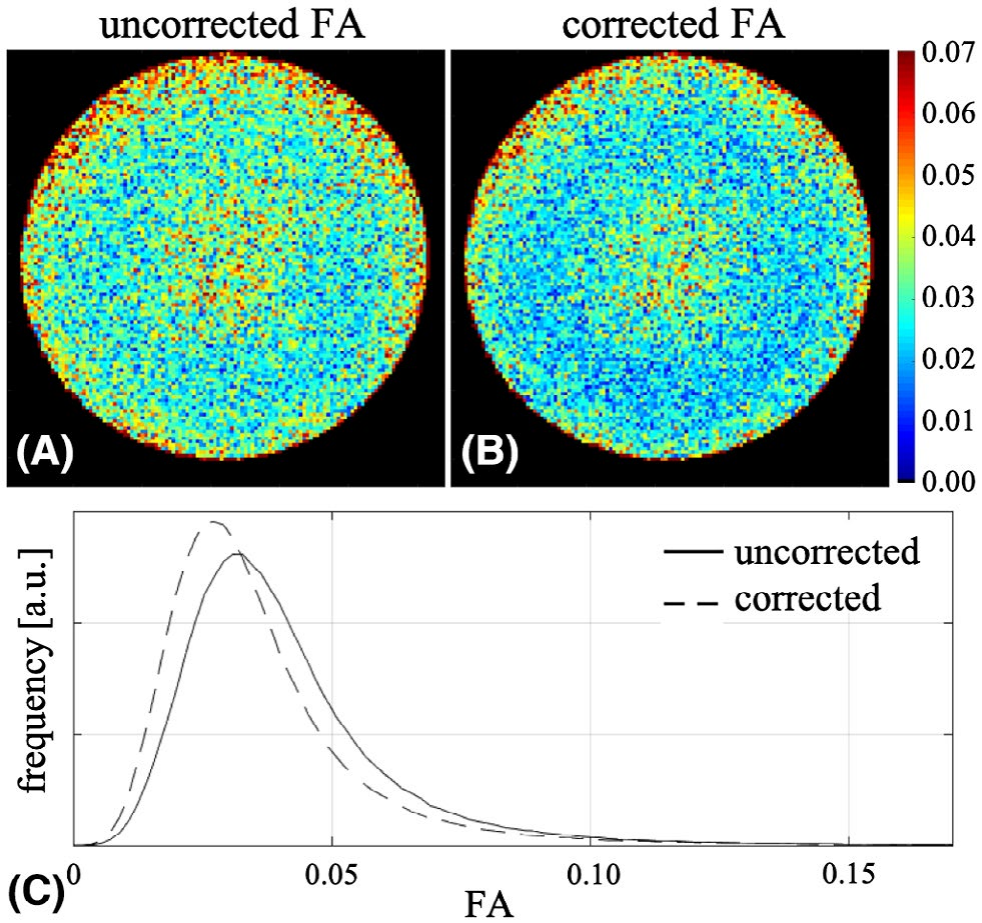
A,B, Central axial slice of the fractional anisotropy (FA) maps without (A) and with (B) voxel‐wise b‐value correction. C, Histograms of the FA values inside the water phantom with (dashed line) and without (solid line) voxel‐wise b‐value correction

The FA values estimated from the 11th HARDI data set acquired on the large water phantom decreased after the voxel‐wise b‐value correction (Figure 3).

**Figure 4:**
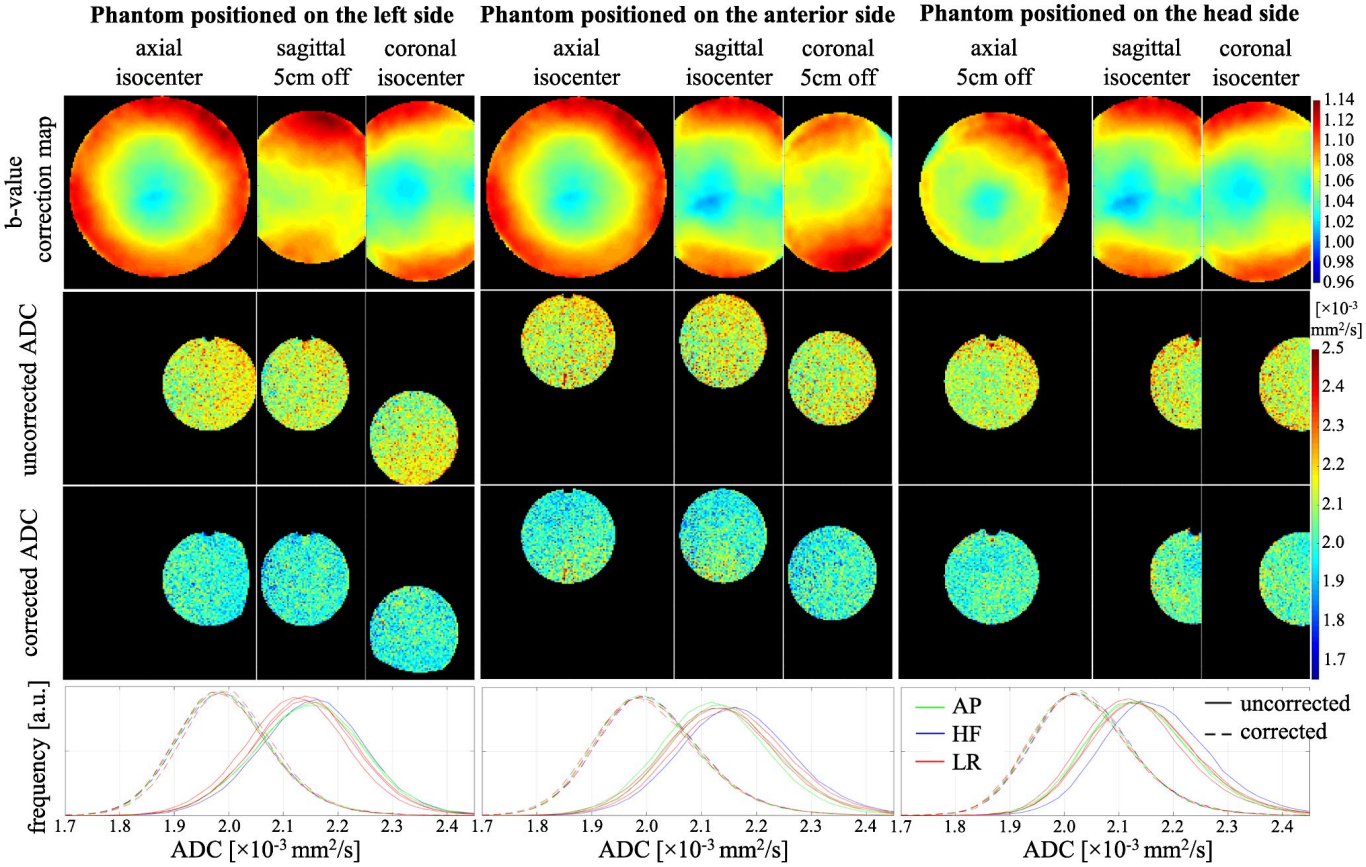
The ADC maps of the small water phantom positioned approximately 5 cm left, anterior, and superior from the isocenter, either without correction (second row) using the nominal b‐value for all voxels or with correction (third row) using the measured b‐value correction map in Experiment_1 (first row). The central slices of the phantom are displayed in all 3 orthogonal orientations for diffusion direction [−0.58, −0.63, −0.51]. Some parts of the phantom were cut off after the correction due to the absence of the measured b‐value. The bottom row shows the histograms of the ADC maps for the three different positions calculated with the nominal (solid lines) or measured (dashed lines) b‐value

The ADC maps of the small water phantom in Experiment_4 had better spatial uniformity across the phantom for all 3 positions (third row in Figure 4) when calculated using the voxel‐wise measured b‐value from Experiment_1 (first row in Figure 4) than globally the nominal b‐value (second row in Figure 4). This was also confirmed by the narrower histograms of corrected ADC values (dotted lines) in comparison to uncorrected values (solid lines). Furthermore, the histograms of corrected ADC values were more similar across 6 diffusion directions than those of the uncorrected ADC maps.

**Figure 5:**
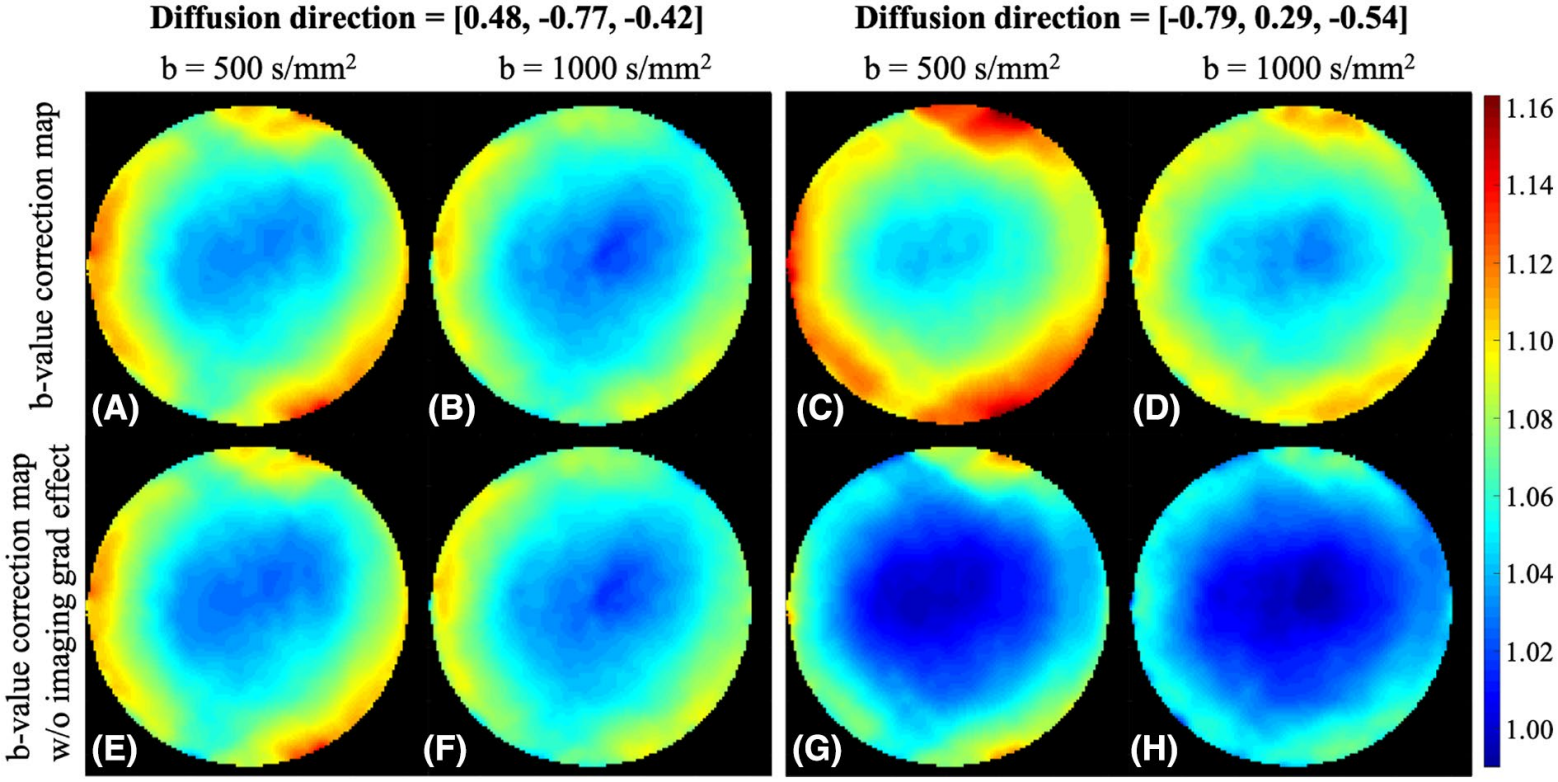
Top row: b‐value correction maps measured at b = 500 s/mm^2^ (A,C) and 1000 s/mm^2^ (B,D) for the two diffusion directions. Bottom row: b‐value correction maps for the same diffusion‐encoding directions are given after removing the imaging gradient effects

There was a discrepancy between b‐value correction maps measured with different nominal b‐values (i.e., 500 s/mm^2^ [Figure 5A,C] and 1000 s/mm^2^ [Figure 5B,D] in Experiment_5). Partially, this discrepancy arises from the imaging gradients, as their influence on the b‐value is not expected to increase with the nominal b‐value. After removing imaging gradient effects from the b‐value correction map, the discrepancy was reduced but still noticeable (Figure 5E‐H). The effects of the imaging gradients on the b‐value deviation were dependent on the diffusion gradient direction, which can be seen by comparing Figure 5A,E against Figure 5C,G.

**Figure 6:**
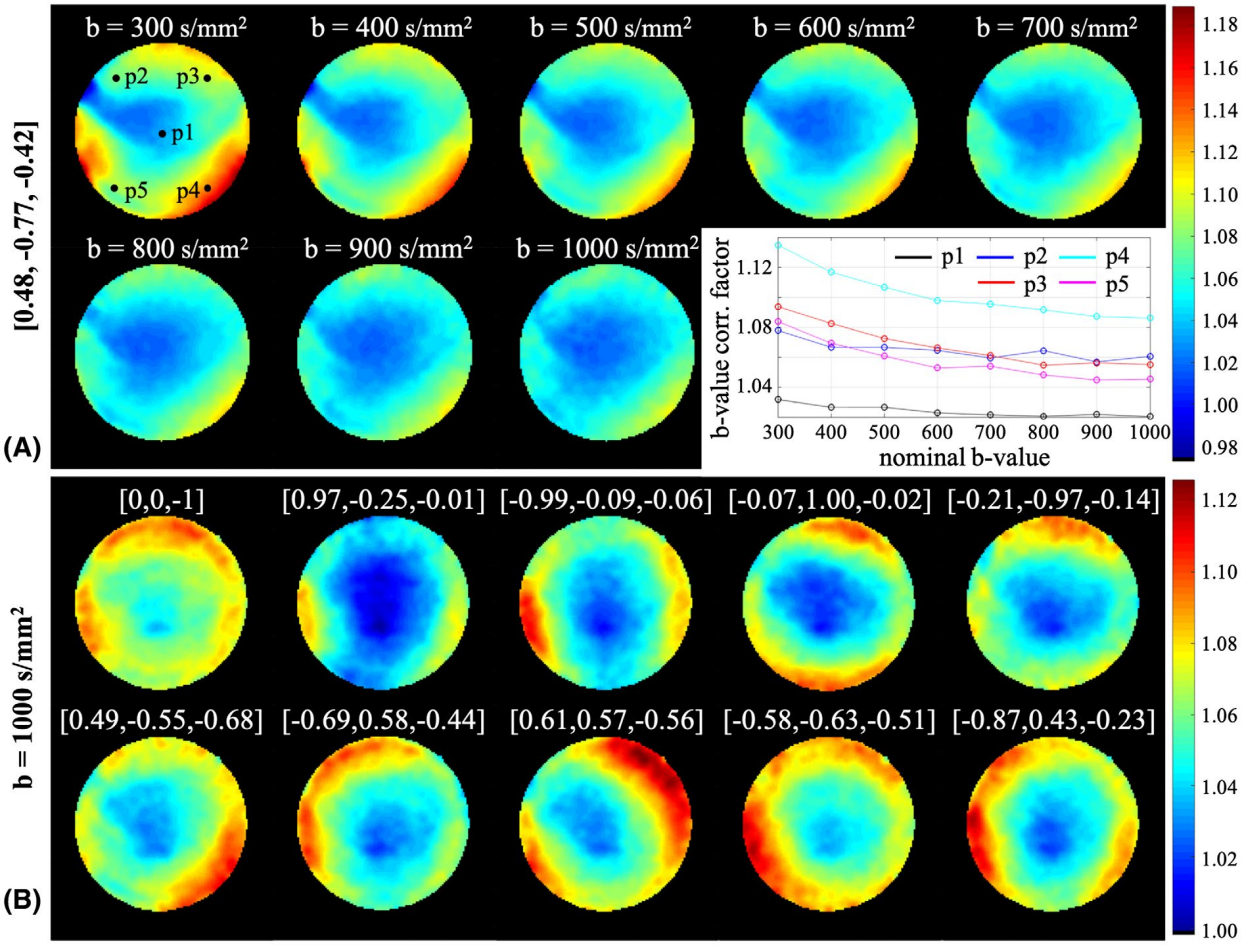
A, Axial slice (5 cm away from the isocenter) of the b‐value correction maps measured at the nominal b‐values between 300 and 1000 s/mm^2^ with the step size of 100 s/mm^2^ for the diffusion direction = [0.48, −0.77, −0.42]. The inset on the bottom right shows the b‐value correction factor as a function of nominal b‐value at 5 different spatial locations. B, Same axial slice of the b‐value correction maps for 10 of 64 diffusion directions at b = 1000 s/mm^2^

The tendency of decreasing b‐value correction factor with increasing nominal b‐value was confirmed from the b‐value correction maps sampled more densely along nominal b‐values (300 to 1000 s/mm^2^, step size of 100 s/mm^2^; Figure 6A). The b‐value correction factors as a function of nominal b‐value at 5 different spatial locations are plotted in the inset of Figure 6A. Each curve exhibited a different slope.

The b‐value correction map varied depending on the diffusion direction (Figure 6B). When the diffusion direction was aligned with the head–foot direction (perpendicular to the plane of displayed images), the axial slice of b‐value correction map had a circular shape (first map in the upper row of Figure 6B). For diffusion encoding along left–right or anterior–posterior direction (left‐right and up‐down respectively in displayed images), oval shapes were observed with the long axis along the direction orthogonal to the corresponding diffusion direction (second to fifth maps from the left in the upper row of Figure 6B). Those patterns were in line with the b‐value map estimated from the spherical harmonic expansion of the magnetic fields.^13^

**Figure 7:**
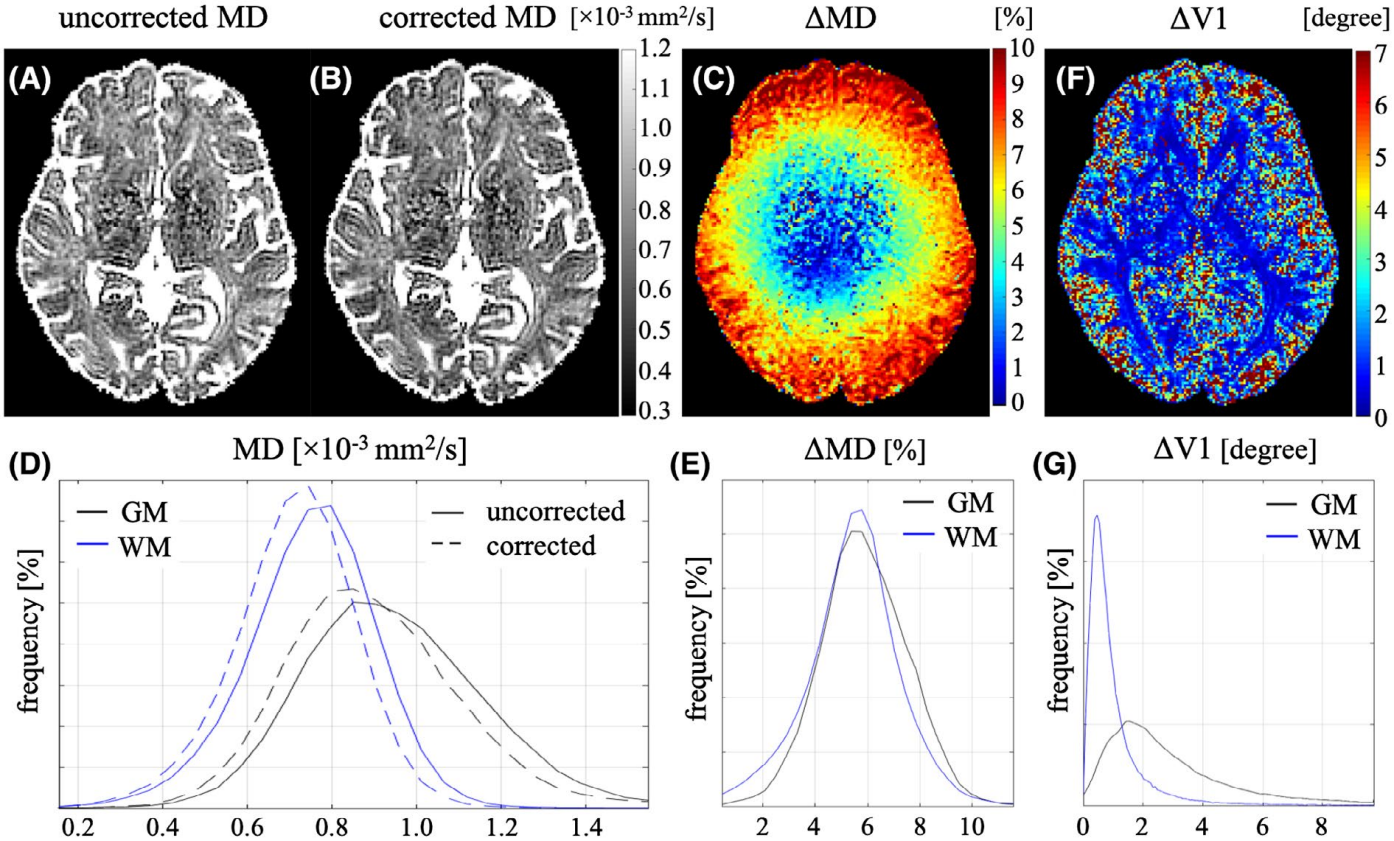
A‐C, Mean diffusivity (MD) maps without (A) and with (B) b‐value correction and their percent difference (C) calculated by ΔMD = 200 × (uncorrected MD – corrected MD) / (uncorrected MD + corrected MD). D, Histograms of MD with (dashed lines) and without (solid lines) b‐value correction inside gray matter (GM; black) and white matter (WM; blue). E, Histograms of ΔMD inside GM (black) and WM (blue). F, Angular difference map between the first eigenvectors of the diffusion tensors estimated with and without b‐value correction calculated by ΔV1 = cos^−1^(**V1**_uncorr_ · ${V1}_{\text{corr}}^{T}$), where **V1** = [V1_x_ V1_y_ V1_z_] is the eigenvector corresponding to the largest eigenvalue of the diffusion tensor, and **V1^T^** denotes its transpose. G, Histograms of ΔV1 inside GM (black) and WM (blue)

For the in vivo data in Experiment_7, although the uncorrected (Figure 7A) and corrected (Figure 7B) mean diffusivity (MD) maps look similar for the chosen scaling and monochrome color map, their relative deviation in percent (Figure 7C) demonstrated a significant difference in the expected pattern. The difference increased with distance from the isocenter. Median values of relative deviations between corrected and uncorrected MD values in GM and WM were 5.8% and 5.5%, respectively (Figure 7E). Moreover, the MD values became more similar after the correction inside both GM and WM (Figure 7D).

The first eigenvector (V1) of the diffusion tensor, estimated with and without b‐value correction, also showed angular discrepancies (Figure 7F,G). As shown in the histograms (Figure 7G), these differences were smaller for WM (median of 0.73°) than for GM (median of 2.71°).

## 4 DISCUSSION

We proposed a simple model‐free approach for correcting the voxel‐wise bias in b‐values and validated it for a diffusion data set at b = 1000 s/mm^2^. The proposed method improved the consistency in ADC values of a water phantom both spatially and across different diffusion directions. In the in vivo study, it was found that the proposed method yielded approximately 5.8% and 5.5% lower MD values in GM and WM, respectively. The primary direction of the diffusion tensor changed 2.71° and 0.73° in GM and WM, respectively, depending on whether the tensor was calculated with the nominal or measured voxel‐wise b‐values.

Overall, the spatial pattern of the experimentally measured b‐value deviation map was in line with the deviation expected from the nonlinearity of the gradient, as predicted through the spherical harmonic expansion of the magnetic fields provided by the vendor. Nonetheless, the measured b‐value maps had larger deviations from the nominal b‐value and more complex spatial patterns. These discrepancies indicate that gradient nonlinearities are not the only source of error in the actual b‐value. Rather, there are further sources of magnetic field gradient deviations, including, but not limited to, concomitant fields, eddy currents, and gradient calibration settings. It is important to note that b‐values scale with the square of the gradient strength. Therefore, even if the errors in the individual sources are small, they can easily add up to a significant overall bias. Concomitant fields are expected to have only a minor impact on b‐value deviations in our data sets for two reasons^17^: (1) the actual impact of concomitant fields on b‐values was reported to be negligible, and (2) the Stejskal‐Tanner diffusion encoding gradients were shown to be insensitive to both the in‐plane and through‐plane dephasing originated from the concomitant fields of the diffusion‐encoding gradients that would otherwise contribute to the measured b‐value deviation. However, this is only true for perfectly symmetric diffusion gradients. If, for example, eddy currents from the first diffusion‐encoding gradient perturb the second diffusion‐encoding gradient in such a way as to break the symmetry, the error would increase.

Ignoring the eddy currents caused by diffusion‐sensitizing gradients in the b‐value calculation would lead to erroneous b‐values, although the errors are expected to be small. In addition, eddy currents may cause small shifts of the refocused slice as compared with the excited slice, which could lead to diffusion direction–dependent signal variations. These signal variations are expected to be reproducible, and therefore the proposed method could correct for them.

Apart from gradient nonlinearities, imaging gradients were another significant source of error in b‐values. After removing the imaging‐gradient effects, the measured b‐value deviation (bottom row in Figure 5) corresponded better to the predicted b‐value deviation from gradient nonlinearities (Figure 1). However, the impact of the imaging gradients varied across diffusion directions due to the interactions between imaging gradients and diffusion gradients (e.g., the diffusion direction [−0.79, 0.29, −0.54] [Figure 5C,D,G,H) was influenced more by the imaging gradients than the diffusion direction [0.48, −0.77, −0.42] [Figure 5A,B,E,F]).

The HARDI data set measured with the nominal b‐value of 1000 s/mm^2^ provided smaller b‐value correction factors than that with the nominal b‐value of 500 s/mm^2^, although the overall spatial patterns were similar (first row of Figure 5). Considering that the b‐value correction factor is calculated by dividing the effective by the nominal b‐value (Equation 4), the smaller b‐value correction factor with b = 1000 s/mm^2^ means that at least some portion of the b‐value deviation is not proportional to the nominal b‐value. Obviously, the imaging gradient interactions would fall in this category. Moreover, gradient coils may behave differently and produce different spatial patterns of gradient nonlinearities, depending on the regime they operate in (e.g., high‐strength or low‐strength regime). Hence, we recommend acquiring the b‐value correction map for each nominal b‐value to achieve a reliable correction. The issue here is that the measurement of the b‐value correction map for a higher b‐value requires more repetitions due to the correspondingly decreasing SNR. For the nominal b‐value of 1000 s/mm^2^ exploited in our experiments, the HARDI acquisitions were repeated 10 times and averaged for increased SNR. However, we found that 92% of the voxels had less than 0.2% difference in b‐value correction factor at the fifth acquisition compared with the fourth acquisition. Thus, 4 repetitions would have sufficed for b = 1000 s/mm^2^. It is noteworthy that we used an 8‐channel head coil and a clinical gradient system with maximum gradient amplitude and slew rate of 40 mT/m and 200 mT/m/ms, respectively. A receive RF coil with increased sensitivity and/or a more advanced gradient system yielding shorter TE would increase the SNR and reduce the required number of repetitions. Nonetheless, for an extremely high b‐value, the number of repetitions required could be impractical. In such a case, a substance with a lower diffusion constant than water^29^, ^34^, ^35^ may be used.

In a previous work,^36^ we presented two ways to obtain the true ADC that is used to calculate b‐value correction factors with Equation 4: (1) using the temperature monitored during the experiment to estimate the true diffusion coefficient of water, and (2) averaging the ADC values around the isocenter. The second approach assumes that the ADC at the isocenter is the true ADC, which holds true only if the gradients can be assumed to be linear at the isocenter and if nonlinearities are the only source of b‐value deviations. However, because we showed here that the imaging gradients play a significant role, yielding b‐value deviations from the nominal b‐value even at the isocenter, we recommend the approach in which an accurate measurement of the temperature is used for reliable quantification of the true ADC. Note that orientational measures such as V1 and FA are not sensitive to the error in the temperature measurement. Although we monitored the temperature with the sensor attached to the surface of the water phantom, we ascertained that making the temperature measurement inside the water itself resulted in a negligible difference.

The proposed method relies on the assumption that the magnetic field imperfections (e.g., gradient nonlinearities, imaging gradient interactions) and their contribution to b‐value deviations are independent of the object being imaged (e.g., water phantom, human brains). As in Experiment_1 and Experiment_4, the ADC values became more spatially homogeneous and consistent across the diffusion directions after the b‐value correction; this assumption appears to be valid. The estimated voxel‐wise ADC of the in vivo data in Experiment_6 further supports this assumption, because after the correction the MD maps became more spatially uniform (Figure 7A‐C). However, a shift of the ADC histograms was observed between scans (Figures 2 and 4). Because shifts were global for all diffusion directions, they may be caused by different scanner calibration settings between scans. These variations from scan to scan exist in all diffusion data sets, making other b‐value correction methods similarly vulnerable. For example, the b‐value correction method based on the spherical harmonic expansion of the magnetic fields suffers from the same issue (data not shown).

Unlike the previous approaches,^13^, ^14^, ^18^, ^20^ the proposed method achieves the b‐value correction without introducing any model to describe magnetic field deviations. Thus, it enables the measurement and correction of the effects that would otherwise be difficult to model with a small number of basis functions or variables. It also allows for capturing effects that are not simply proportional  to the nominal b‐value (Figure 5).

There are some limitations to the proposed correction method. First, being image‐based, it is inherently sensitive to image artifacts such as EPI ghosting and distortions due to magnetic susceptibility effects and eddy currents. To minimize these artifacts we used magnetic field monitoring technologies and subsequently performed off‐line image reconstruction with this additional information^24^, ^26^, ^27^, ^28^ for both the phantom and in vivo data. Second, it may be considered a limitation that the proposed method cannot correct the deviations in gradient orientation, whereas other, model‐based approaches provide an avenue for correcting the deviations in both the b‐value and the diffusion‐encoding direction.^13^ However, because the latter method considers a single source of deviation (i.e., gradient nonlinearity), it cannot capture other sources of error and the detailed spatial deviation they cause in the vowel‐wise b‐value (Figure 1). Whether it is better to partially correct both the b‐value and the diffusion‐encoding directions, or make a more comprehensive correction of only the b‐value, is difficult to ascertain and will have to be addressed in future work. Third, the proposed method neglects the diffusion‐encoding effects of the imaging gradients in the b_0_ image. For a truly quantitative measurement of the diffusion constant, the diffusion encoding of the b_0_ image would have to be included. This effect was ignored, as it is very small (1.08 s/mm^2^ for b = 1000 s/mm^2^) compared with the significant improvement from the proposed correction in its present form. Fourth, the b‐value correction is possible inside the volume covered by the phantom used for the measurement of the b‐value correction map. The water phantom used here had an inner diameter of 19 cm and was large enough to cover the brain. Making sure that both the water phantom and the participant are scanned at the center of the head coil can help to ensure that the water phantom appropriately covers human brains. Finally, the presented method requires that the b‐value correction map and in vivo data are collected with the same sequence. Although a wide range of b‐values are used in neuroimaging, this manuscript demonstrated the proof‐of‐principle at a b‐value of 1000 s/mm^2^.

## 5 CONCLUSIONS

We introduced a novel approach to obtain the effective b‐value map from phantom diffusion data and used this map to estimate more reliable diffusion‐related measures. This approach is simple and easy to perform, even without any prior knowledge of the gradient field distributions generated by each gradient coil, which not all vendors provide to all sites. The proposed b‐value correction holds a potential in both research and clinics, especially in multicenter settings, where the spatial pattern of the b‐value map will vary among the sites with different scanner models.^20^

## CONFLICT OF INTEREST

K.P.P. holds a research agreement with and receives research support from Philips. He is a shareholder of Gyrotools and Skope Magnetic Resonance Technologies. B.J.W. is a shareholder and part‐time employee of Skope Magnetic Resonance Technologies.
